# Wild *Vicia* Species Possess a Drought Tolerance System for Faba Bean Improvement

**DOI:** 10.3390/genes13101877

**Published:** 2022-10-17

**Authors:** Irfanul Haq, Dalal Nasser Binjawhar, Zahid Ullah, Ahmad Ali, Hassan Sher, Iftikhar Ali

**Affiliations:** 1 Center for Plant Sciences and Biodiversity, University of Swat, Charbagh Swat 19120, Pakistan; 2 Department of Chemistry, College of Science, Princess Nourah bint Abdulrahman University, Riyadh 11671, Saudi Arabia; 3 State Key Laboratory of Molecular Developmental Biology, Institute of Genetics and Developmental Biology, Chinese Academy of Sciences, Beijing 100101, China

**Keywords:** *Vicia* wild relatives, faba bean, drought stress, vascular anatomy, antioxidants

## Abstract

Faba bean (*Vicia faba* L.), a drought-sensitive crop, is drastically affected by drought stresses compromising its growth and yield. However, wild relatives of faba bean are considered a reservoir of potential genetic resources for tolerance against abiotic stresses. This study was conducted to characterize wild relatives of faba bean for identification of a specific tolerance system required for its improvement against drought stress. The study focused on physiological, biochemical, and anatomical responses of wild *Vicia* species under drought stress conditions. The experiment was carried out under various levels of drought stress imposed through different field capacities (FC) which included 80% FC ie (well-watered condition), 55% FC (moderate stress), and 30% FC (severe stress). When compared to plants grown in a control environment, drought stress significantly reduced the studied physiological attributes including soluble sugars (21.3% and 15.8%), protein contents (14.7 and 14.6%), and chlorophyll (8.4 and 28.6%) under moderate (55% FC) and severe drought stress (30% FC), respectively. However, proline content increased by 20.5% and 27.6%, peroxidase activity by 48.5% and 57.1%, and superoxide dismutase activity by 72.6% and 64.8% under moderate and severe stress, respectively. The studied anatomical attributes were also affected under drought stress treatments, including diameter of stem xylem vessels (9.1% and 13.7%), leaf lower epidermal thickness (8.05% and 13.34%), and leaf phloem width (5.3% and 10.1%) under moderate and severe stress, respectively. Wild *Vicia* spp. showed better tolerance to water-deficit conditions as compared to cultivated *Vicia* L. The observed potential diversity for drought tolerance in wild *Vicia* spp. may assist in improvement of faba bean and may also help in understanding the mechanisms of adaptations in drought-prone environments.

## 1. Introduction

Among legumes, the genus *Vicia* has remarkable economic importance for humans and animals on account of its food, fodder, and medicinal value [1]. Faba bean (*Vicia* L. Fabaceae) is a cool-season annual legume that is consumed by humans for nutritional purposes as it contains about 35% proteins, in addition to complex carbohydrates, choline, lecithin, and dietary fibers, and because it is a rich source of nutrients such as calcium, magnesium, potassium, and zinc [2,3]. Moreover, the seeds of faba bean have lower amounts of cholesterol, fats, and sodium [4]. Its seeds are routinely used as a substitute of fish and meat proteins in the developing countries of Asia and Africa [5]. It also plays an important role in increasing soil fertility by fixing atmospheric nitrogen and solubilizing phosphorus (P) in the soil, enhancing plant–microbe interactions [6,7]. Medicinally, faba bean is valuable in accumulating a substantial concentration of *L*-Dopa, a precursor of dopamine used for treating hormonal imbalance [3,8]. Various species of *Vicia* possess antioxidant, antimicrobial, anti-Parkinson, and antidiabetic potentials [9]. Its annual total production has been estimated as 14.2 Mt on cultivated land of 13.7 Mha; however, there are reports of a decreased global acreage and yield of faba bean since 1980 [10,11,12,13]. The yield of *Vicia* L. is reduced by deficiency of water, subjected at any stage of the life cycle; however, flowering, podding, and grain-filling phases are the most sensitive [14,15,16]. Further, different varieties of faba bean may have differential responses toward abiotic stresses [17,18]. To adopt plants with deficient water conditions, plant breeders and physiologists are trying to improve the phenology and physiology of crops by developing drought-tolerant cultivars [19,20,21]. However, improvement in the development of cultivars for drought tolerance is slow because of a lack of efficient screening techniques and large seasonal variation [22,23,24,25].

Crop wild relatives (CWRs) can play important roles in the improvement of cultivated crops because of exhibiting resistance to various abiotic and biotic stresses [26,27,28]. Different genes which are present in CWRs can be utilized to develop new varieties which produce better yield and tolerance [29]. For instance, a few genes reported in *Oryza nivara,* a wild relative of rice, have shown resistance to various plant diseases [30]. Hence, different genes can be isolated from CWRs and transferred to cultivated crops to achieve high yields, because they possess resistance to abiotic and biotic stresses [31].

Vetches are mostly grown in the wild and are found naturally in rain-fed conditions [29]. *Vicia* is a taxonomically large and complex genus represented by about 210 to 240 species, distributed primarily in temperate regions of the northern hemisphere in Asia, Europe, and North America, and extending into extratropical South America as well [32]. Mediterranean and Irano-Turanian regions are considered the center of origin and diversity for the genus *Vicia* [33]. *Vicia sativa* subsp. *sativa* L. can grow in humid as well as in semi-dry areas and is used as livestock feed. *Vicia villosa* subsp. *dasycarpa* Roth. is a cool-season legume, grown for pasture, hay, silage, and grains for livestock. *Vicia narbonensis* L. is also a cool-season annual legume, having the potential to produce more grains in non-tropical areas in comparison to *Vicia sativa*, *Vicia ervilia,* and *Vicia villosa* subsp. *dasycarpa* [34]. Members of the *Vicia* genus have medicinal importance and also fix atmospheric nitrogen to a usable form and hence increase the fertility of soil [35,36].

The production of the legumes crop is mostly affected by various abiotic stresses such as cold, salinity. and drought, which cause a reduction in yield in different parts of the world [37]. Drought stress is a major abiotic stress which affects the yield of cereals, faba bean, and other legumes. It affects various physiological processes of plants such as cell wall turgidity, reduced carbon assimilation rate, and increased oxidative changes, the result being decreased yield [38,39]. Further, it also affects flowering times in plants [40,41]. Due to drought stress, metabolic activities of plants may lead to the formation of reactive oxygen species (ROS) which cause the oxidation of organic molecules such as DNA, RNA, lipids, and proteins [42,43]. After thorough review, it was revealed that there is no work reported to date on the biochemical and anatomical characterization of wild *Vicia* spp. of the study area. Therefore, the present work aimed to evaluate selected wild relatives of faba bean under progressive drought stress. The main focus was to investigate their physiological, biochemical, and anatomical responses under water-deficit conditions and to explore potential *Vicia* spp. with future prospects in breeding for drought tolerance.

## 2. Materials and Methods

### 2.1. Plant Specimen and Seed Collection

The genus *Vicia* is represented in Pakistan by 14 species, mostly confined to the northern temperate regions of Gilgit-Baltistan, Khyber Pakhtunkhwa, Upper Punjab, and Northern Baluchistan. Of these, *V. faba* L. is known only in cultivation, while the rest of the species are wild, or have become naturalized (*V. narbonensis*, *V. villosa*). During the fieldwork, about 450 specimens belonging to *Vicia sativa* subsp. *sativa*, *V. sativa* subsp. *nigra, V. narbonensis, V. bithynica,* and *V. monantha* were collected from different localities in twelve districts of Northern Pakistan including Swat, Dir, Bajour, Chitral, Mansehra, Battagram, Abbotabad, Islamabad, Skardu, Gilgit, Muzaffarabad and Neelum valley Azad Jammu, and Kashmir. Collections were done during flowering and fruiting periods of the species from March to September 2019 in different ecological habitats. Sampling was done randomly, and at least five specimens per species were sampled in each population in different areas, and, in total, more than sixty populations were sampled. The majority of the sampling was done in Swat, followed by Chitral and Mansehra. During fieldwork information on locality, phenology, population size, habitat features, number of flowers, number of pods, and seeds per pod were noted in a field notebook. A specific collection number was assigned to each specimen and field photographs of the whole plant and plant parts were taken. The specimens were dried, preserved, and mounted on herbarium sheets and submitted as vouchers to the Herbarium University of Swat (SWAT). Specimens were identified to species and subspecific levels by the second author using the flora of Pakistan and flora of Iran [44,45]. The nomenclature of scientific names is in accordance with Kew database Plants of the World Online “https://powo.science.kew.org/ (accessed on 23 June 2021)” and World Flora Online database “http://www.worldfloraonline.org/ (accessed on 23 June 2021)”. Seeds were collected from mature pods during May and September in cotton bags, dried, and stored in dark sealed bottles. Seeds of cultivated faba bean were provided by NARC (National Agriculture Research Centre) Islamabad. Species used during the current study included *Vicia sativa* ssp. *Sativa* L., *V. sativa* ssp. *Nigra* Ehrh., *V. narbonensis* L., *V. bithynica* (L.) L., *V. faba* L., and *V. monantha* Retz.

### 2.2. Pot Experiment

An experiment was carried out in green house at the Center for Plant Sciences and Biodiversity, University of Swat, for comparing the physiological, biochemical, and anatomical responses of the experimental plant materials comprising of wild *Vicia* species under drought stress conditions. Seeds were germinated in petri plates, and healthy seedlings were transplanted into plastic pots filled with soil and exposed to sunlight for better growth. The field capacity (FC) was determined gravimetrically following the method of Liu and Li [46] and Graber et al. [47]. Treatments in the pot experiment included 80% FC ie (well-watered condition), 55% FC (moderate stress), and 30% FC (severe stress). The experimental design employed during the study was a randomized complete block design (RCBD) replicated three times. To monitor drought stress, each pot was weighted daily, and water lost through evapotranspiration was added to pots. Thirty days after the imposition of stress, leaf samples were collected, weighted, wrapped in aluminum foil, and kept in a refrigerator at 40 °C for the determination of various anatomical, physiological, and biochemical attributes as described in Ali et al. [23].

### 2.3. Leaf Epidermal Anatomy

For leaf epidermal anatomy, fresh leaves were collected and boiled in 88% lactic acid for 30–40 min at 100 °C in a water bath according to the method used by Clark [48] and Ullah et al. [49]. To attain the peel of the abaxial surface of leaf, the adaxial surface was removed, along with mesophyll tissues by using a sharp blade, leaving the peel of the abaxial surface. To attain the peel of the adaxial epidermis, the same procedure was repeated. The leaves were softened with lactic acid, and the abaxial and adaxial peels were removed using a sharp razor blade. Epidermal peels were stained with safranin and mounted on glass slides in lactic acid. The studied qualitative traits were the epidermal cell shape, wall morphology, and stomata types, while quantitative traits included the subsidiary cell number and shape, length, width, and abundance of stomata, as well as the trichome type and size examined by using a light microscope equipped with a digital camera (Meiji infinity DK-5000, Tokyo, Japan).

### 2.4. Anatomical Features of Leaf and Stem

The anatomical study of leaves and stems was carried out to study variations in different traits in response to drought stress according to Ruzin [50]. Small pieces of plant tissue were fixed for 24 h in formalin acetic alcohol (FAA) solution containing formalin (10 mL), 70% ethyl alcohol (85 mL), and glacial acidic acid (85 mL). Slides were prepared by free-hand sectioning using sharp blades. Sections were then dehydrated by ethanol series (30%, 50%, 70%, and 90%). For clear visibility, sections were stained with light green and safranin, which were studied under light microscopes. Different anatomical features of stems and leaves such as epidermal cell length and width, stomatal length and width, stomatal complex length and width, trichome length and width, phloem length and width, and metaxylem diameter were recorded accordingly.

### 2.5. Physiological and Biochemical Analysis

Chlorophyll contents were determined according to the method used by Hiscox and Israelstam [51] using dimethyl sulfoxide (DMSO). Proline contents were determined in leaf samples using 3% sulfosalicylic acid by following the method of Bates et al. [52]. Protein contents in leaves were determined by following the method of Lowery et al. [53], using bovine serum albumin (BSA) as a standard. Soluble sugar contents were determined according to the method used by Dubois et al. [54], using the phenol sulfuric acid method, with a little bit of modification. For the determination of superoxide dismutase (SOD) activity, the method of Beauchamp and Fridovich [55] was followed, and peroxidase (POD) activity was accomplished according to Gorin and Heidema [56].

### 2.6. Statistical Analysis

For statistical analysis, the data of samples were recorded in Microsoft excel. The experimental design employed was a two-factor factorial RCBD (randomized complete block design) with three replications. The two treatment factors were six *Vicia* species and three drought stresses. Quantitative data were shown by drawing graphs and were also subjected to an analysis of variance (ANOVA) and least significant differences using Statistix 8.1., Tallahassee, FL, USA. Comparison among treatment means was made as described in Ali et al. [42]. For graphical representation of the association among different studied traits, the data were subjected to a Pearson correlation coefficient and principle component analysis by employing the Jamovi software.

## 3. Results

### 3.1. Leaf Micromorphological Attributes

**Figure 1:**
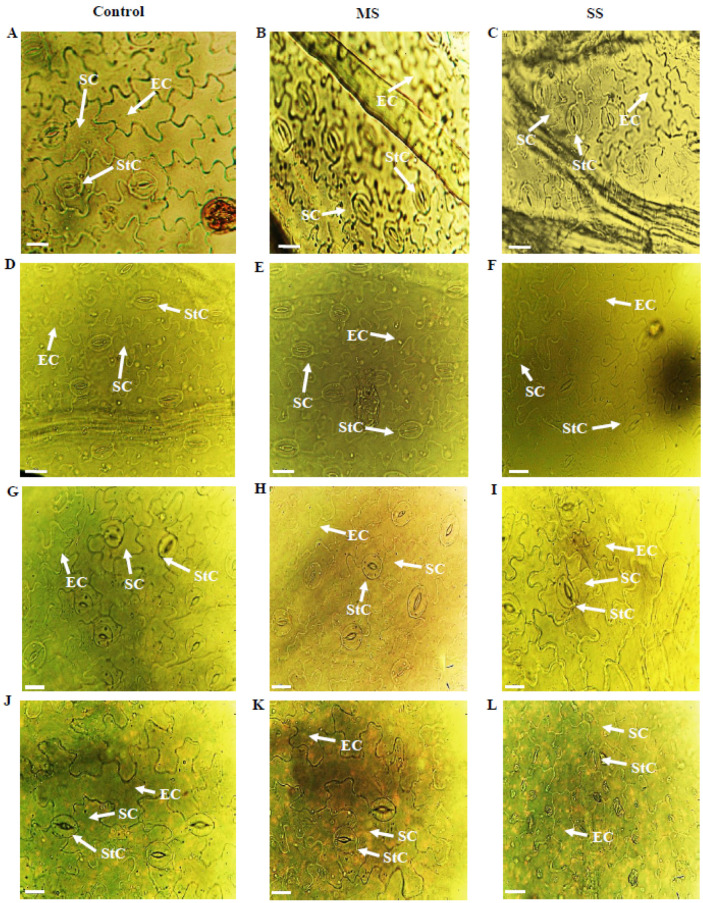
Epidermal anatomy of abaxial (AB) surface of *Vicia species* showing variations in epidermal cells (EC), stomatal cells (SC) and stomatal complexes (StC). (**A**) *V. sativa* subsp. *sativa* control, (**B**) *V. sativa* subsp. *sativa* 55% FC, (**C**) *V. sativa* subsp. *sativa* 30% FC, (**D**) *V. sativa* subsp. *nigra* control, (**E**) *V. sativa* subsp. *nigra* 55% FC, (**F**) *V. sativa* subsp. *nigra* 30% FC, (**G**) *V. narbonensis* control, (**H**) *V. narbonensis* 55% FC, (**I**) *V. narbonensis* 30% FC, (**J**) *V. bithynica* control, (**K**) *V. bithynica* 55% FC, (**L**) *V. bithynica* 30% FC. Bar scale 20 μm.

Results showed that both moderate and severe drought stress significantly affected various biochemical and physiological characteristics of *Vicia* spp. Mean square results showed that *Vicia* spp. and treatments highly differed for all the studied attributes in abaxial and adaxial leaf surfaces. However, their interaction was different only for stomatal width, while no significant differences were found for the rest of the studied traits (Table 1, Table 2, Table 3, Table 4 and Table 5; Figure 1). Drought stress significantly reduced all the studied attributes of both epidermal surfaces of leaves. Epidermal cell length decreased by 6.1% and 9.4% under moderate and severe drought stress, respectively. Similarly, epidermal cell width declined by 9.9% and 11.2% in moderate and severe stress, respectively. The stomatal length increased by 4.1% under moderate stress and 9.7% under severe stress. Under moderate stress, the stomatal width decreased by 5.8% while the decrease was 11.8% in severe drought stress. Stomatal complex length, trichome length, trichome width, and stomatal complex width also declined in response to progressive drought stress.

### 3.2. Anatomical Attributes

**Figure 2:**
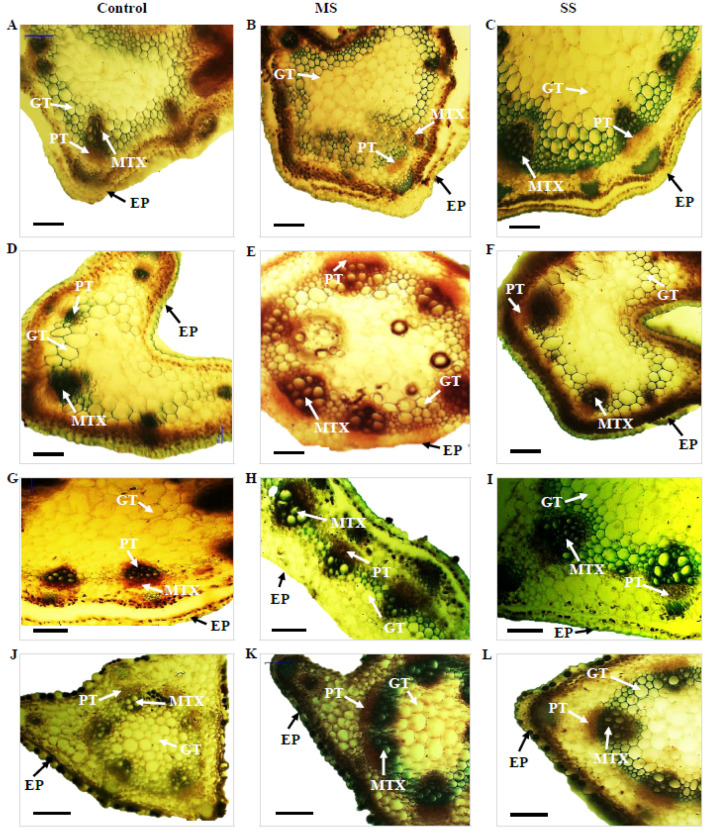

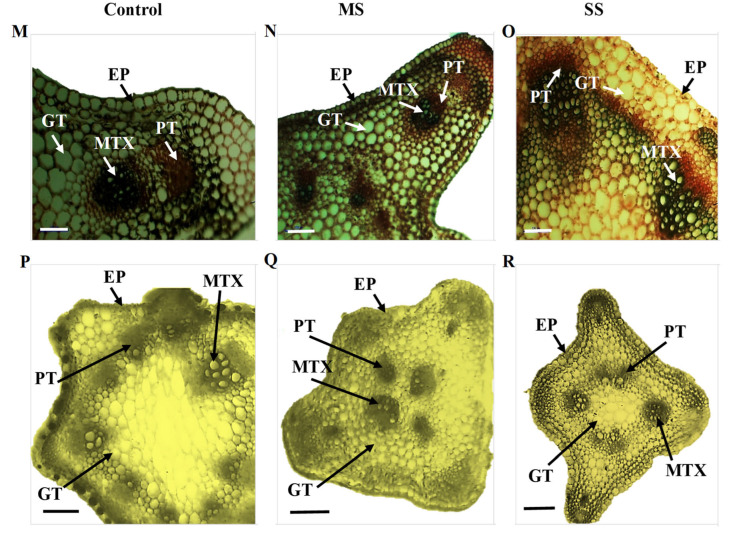
Transverse section of the stem of *Vicia species* showing variation in the studied anatomical traits, i.e., epidermis (EP), metaxylem (MTX), phloem (PT), and ground tissues (GT); (**A**) *V*. *sativa* subsp. *sativa* control, (**B**) *V*. *sativa* subsp. *sativa* 55% FC, (**C**) *V*. *sativa* subsp. *sativa* 30% FC, (**D**) *V*. *sativa* subsp. *nigra* control (**E**) *V*. *sativa* subsp. *nigra* 55% FC, (**F**) *V*. *sativa* subsp. *nigra* 30% FC, (**G**) *V*. *narbonensis* control, (**H**) *V*. *narbonensis* 55% FC, (**I**) *V*. *narbonensis* 30% FC, (**J**) *V*. *bithynica* control, (**K**) *V*. *bithynica* 55% FC, (**L**) *V*. *bithynica* 30% FC, (**M**) *V*. *faba* control, (**N**) *V. faba* 55% FC, (**O**) *V. faba* 30% FC, (**P**) *V. monantha* control, (**Q**) *V. monantha* 55% FC, (**R**) *V. monantha* 30% FC. Bar scale 100 μm.

Mean square results concerning anatomical attributes revealed that epidermis thickness, phloem length, phloem width, and metaxylem diameter were highly different among the studied *Vicia* spp. Similarly, the interaction between *Vicia* spp. and drought stress treatment resulted in high differences in epidermis thickness, phloem length, phloem width, and metaxylem diameter (Table 6). Drought stress significantly reduced all the studied attributes of stem vascular anatomy. For instance, epidermis thickness decreased by 8.8% under moderate and 15.2% under severe drought stress. The decrease in phloem length was also recorded under moderate (3.0%) and severe (6.4%) drought stress. Similarly, phloem width declined by 3.9% under moderate and 8.4% under severe drought stress. Likewise, metaxylem diameter decreased by 9.1% under moderate drought stress and 13.7% under severe drought stress (Table 6; Figure 2). The maximum change (18.9%) in epidermal cells thickness under moderate drought stress was recorded in *Vicia sativa* subsp. *sativa* while minimum change (3.6%) was recorded in *Vicia bithynica*. Similarly, the maximum change (5.3%) in phloem width was recorded in *Vicia* under moderate stress while the minimum change (4.9%) was recorded in *Vicia sativa* subsp. *sativa*. Similarly, the maximum change in metaxylem diameter was recorded in *Vicia bithynica,* while the minimum change was in *Vicia narbonensis* under severe drought stress (Table 6; Figure 2).

### 3.3. Chlorophyll Contents

**Figure 3:**
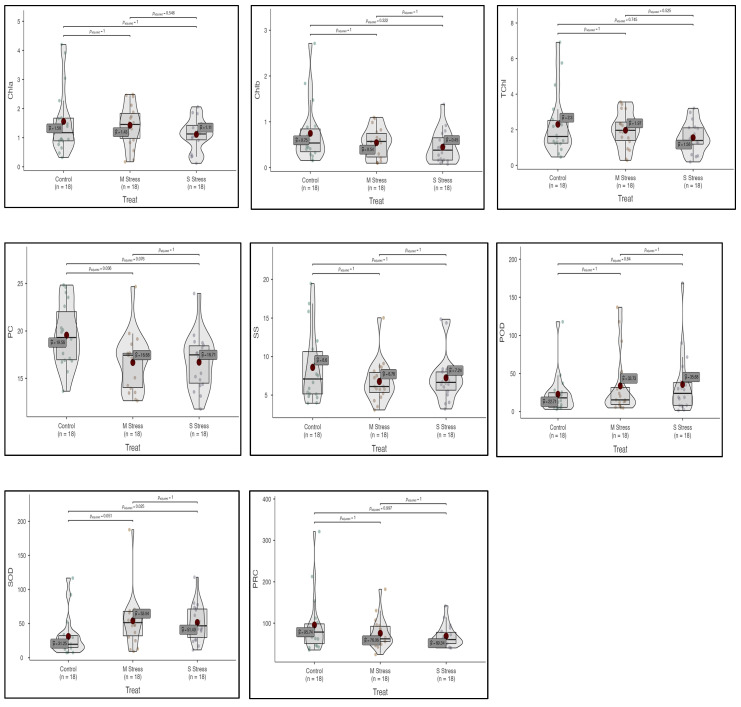
Box–violin plots presenting physiological and biochemical traits, i.e., chlorophyll a (Chla), chlorophyll b (Chlb), total chlorophyll (TChl), protein content (PC), soluble sugars (SS), peroxidase activity (POD), superoxide dismutase activity (SOD), and proline content (PRC) under control (C), moderate (MS) and severe (SS) drought stress conditions.

Photosynthetic pigments were also affected by both moderate and severe drought stress as compared to the control. Chlorophyll a (Chla) content decreased both in moderate and severe stress by (8.4% and 28.6%), respectively. Chlorophyll b (Chlb) contents were decreased by 27.3% and 40.4% in moderate and severe stress, respectively. Similarly, total chlorophyll contents also decreased by 14.4% and 32.4% in both stresses, respectively (ESM 1; Figure 3). Among *Vicia* species, the maximum change in Chla contents was recorded in *V. narbonensis,* while the minimum change was in *Vicia sativa* subsp. *sativa* under moderate and severe drought stress, respectively. Similarly, the maximum change in TChl content was observed in *V. narbonensis,* while the minimum change was in *V. sativa* subsp. *sativa* under moderate and severe stress treatments, respectively (ESM 1, 2; Figure 3).

### 3.4. Biochemical Attributes

Soluble sugar (SS) decreased by 21.3% and 15.8% in moderate and severe stress, respectively. A reduction in protein contents was observed, which was 14.7% and 14.6% in moderate and severe stress, respectively. Proline contents also decreased by 20.5% under moderate drought stress, but increased by 27.58% under severe drought stress. Peroxidase activity (POD) increased by 48.5% and 57.1% in moderate and severe stress, respectively. An increase in superoxide dismutase (SOD) activity was also observed at 72.6% and 64.8% in moderate and severe stress, respectively (ESM 1,3,4; Figure 2 and Figure 3). A maximum change in peroxidase (POD) activity was recorded in *Vicia sativa* subsp. *nigra* under moderate stress, while a minimum change of 12.5% was recorded in *Vicia narbonensis* under severe drought stress (ESM 4; Figure 2).

A maximum change in soluble sugar (SS) was recorded at 55.23% in *Vicia sativa* subsp. *nigra* while a minimum change in SS was recorded at 4.25% in *Vicia bithynica* under severe drought stress. Similarly, a maximum change in protein contents was recorded at 30.94% in *Vicia narbonensis* under moderate stress, while a minimum change was recorded at 3.54% in *Vicia bithynica* in severe drought stress. A maximum change in proline contents under moderate drought stress was recorded at 53.43% in *Vicia sativa* ssp. *Nigra,* while a minimum change in proline contents was recorded 4.57% in *Vicia bithynica* in severe drought stress (ESM 5; Figure 2).

### 3.5. Heatmap of Pearson’s Coefficient

**Figure 4:**
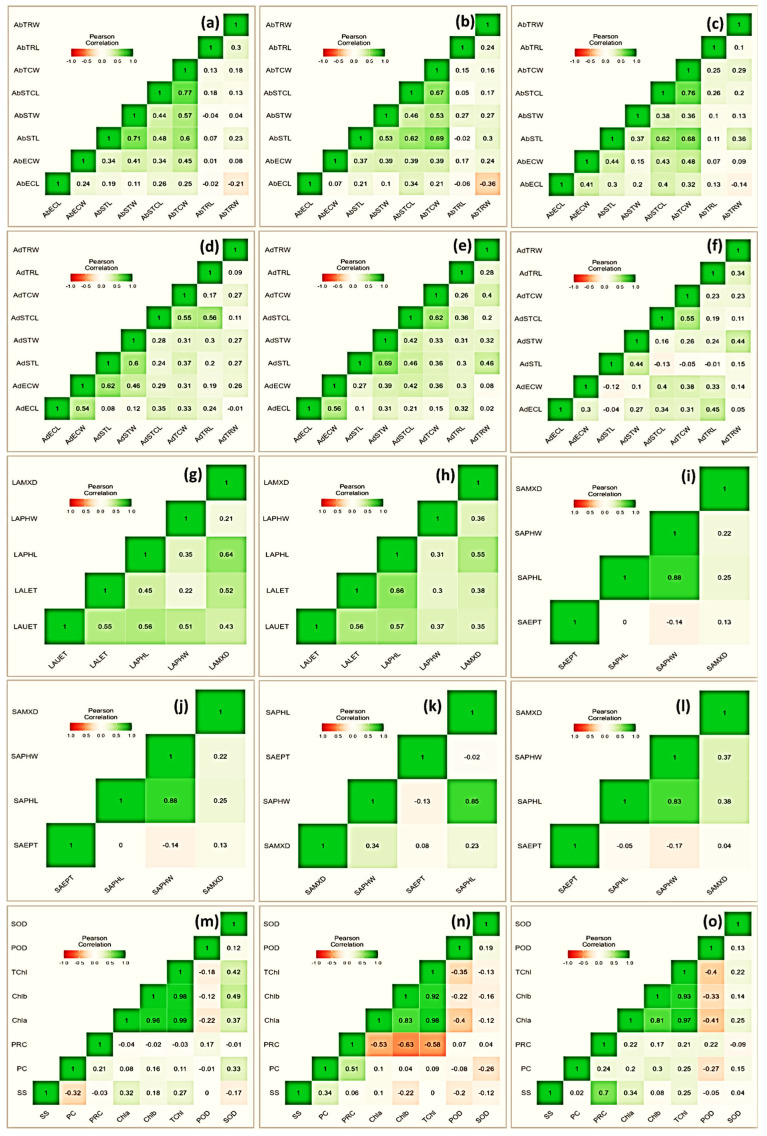
Heatmap showing Pearson correlation coefficient and associated probabilities (*p* ≤ 0.05, ≤ 0.01, ≤ 0.001, respectively) among *Vicia* species for the studied traits (n = 10) evaluated under drought stress (pot experiment with three treatments, i.e., control, FC = 80%; MS, FC = 50%; SS, FC = 30%). Micromorphological traits represent both abaxial and adaxial surfaces, i.e., trichome width (AbTRW and AdTRW), trichome length (AbTRL and AdTRL), stomatal complex length (AbSTCL and AdSTCL), stomatal complex width (AbSTCW and AdSTCW), stomatal length (AbSTW and AdSTW), stomatal width (AbSTL and AbSTL), epidermal cell length (AbECL and AdECL), and epidermal cell width (AbECW and AdECW). Leaf anatomical traits included metaxylem diameter (LAMXD), phloem width (LAPHW), phloem length (LAPHL), lower epidermis thickness (LALET), and upper epidermis thickness (LAUET). Stem anatomical traits included metaxylem diameter (SAMXD), phloem width (SAPHW), phloem length (SAPHL), and epidermis thickness (SAEPT). (**a**) Control, (**b**) MS, and (**c**); SS (**d**) control, (**e**) MS, and (**f**) SS showing relationship for leaf micromorphological traits; (**g**) control, (**h**) MS, and (**i**) SS showing relationship for leaf anatomical traits; (**j**) control, (**k**) MS, and (**l**) SS showing relationship for stem anatomical traits; and (**m**) control, (**n**) MS, and (**o**) SS showing relationship for physiological traits.

A heatmap of Pearson’s coefficient of correlation (r) analysis of the investigated attributes depicted variable associations (Figure 4). Among physiological traits, superoxide dismutase exhibited a significantly positive correlation. Specifically, its association was more prominent with Chla (r = 0.37), Chlb (r = 49), TChl (r = 0.42), and PRC (r = 0.33). The association of proline content was noticeable with chlorophyll with r = −0.53, −0.63 and −0.58 for Chla, Chlb, and TChl, respectively, under a moderate stress environment. The protein content correlated inconsistently with most of the studied traits, especially under stress treatment; however, its association was positive with SS (r = 0.34) and PRC (r = 0.51). Similarly, among leaf micromorphological traits, stomatal length in abaxial surface correlated strongly with stomatal width (r = 0.53 and 0.37), trichome cell length (r = 0.62 and 0.63), trichome cell width (r = 0.69 and 0.68), and epidermal cell width (r = 0.0.37 and 0.41), under moderate and severe drought stress, respectively. Among leaf anatomical traits, phloem length was in positive association with metaxylem diameter (r = 0.55), and upper and lower epidermal thicknesses (r = 0.57 and 0.66, respectively) under moderate drought stress. Further, with respect to the studied stem anatomical traits, the highest positive association was recorded between phloem length and width (r = 0.85 and 0.83) under moderate and severe stress, respectively.

### 3.6. Principle Component Analysis (PCA)

**Figure 5:**
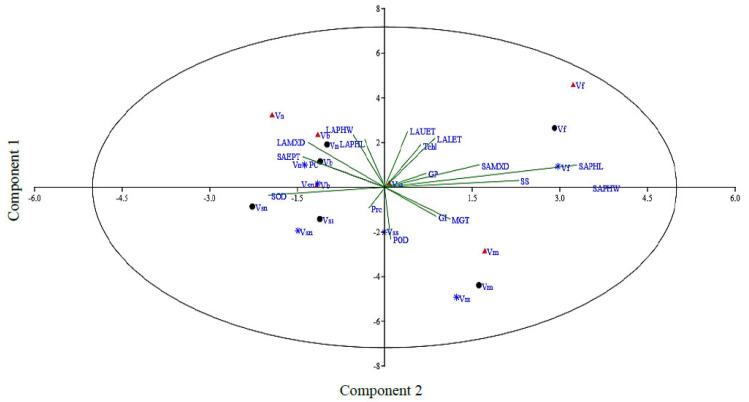
Principal component analysis of the studied attributes in *Vicia* species evaluated under pot induced drought stress (control, FC = 80%; moderate stress, FC = 50%; severe stress, FC = 30%, represented by black, blue, and crimson colors, respectively). Vss, *V*. *sativa* subsp. *sativa;* Vsn, *V*. *sativa* subsp. *nigra*; Vn, *V*. *narbonensis;* Vb, *V*. *bithynica;* Vf, *V*. *faba;* Vm, *V. monantha*.

Principle component analysis can be utilized to select traits that can be categorized into main groups and subgroups based on homogeneity and dissimilarity. In order to find out the most appropriate combination of the studied attributes, PCA and biplot analysis were conducted using mean values (Figure 5 and ESM 5). The vector length shows the extent of variation explained by respective traits in the PCA. The first two axes, i.e., PC1 (eigen value = 6.68) and PC2 (eigen value = 3.24), explained up to 55% of the total variability. Considering PC1 and PC2, it is very clear that mostly morpho-physiological, biochemical, and leaf epidermal attributes contributed to PC1, while the attributes with major contributions to PC2 were mostly leaf epidermal and biochemical traits. The attributes in order of their positive contribution to PC1 included leaf upper epidermis thickness (0.37) and phloem width (0.35). The contribution from the remaining studied attributes to this principal component was either positive or negative but non-significant. Similarly, for PC2, the major contributing attributes were stem phloem width (0.52), phloem length (0.49), and SS (0.34). The contribution of all other studied traits to this principal component was minor. For PC3, stem metaxylem diameter (0.27) was noted for its prominent contribution. For PC4, leaf phloem length (0.38) was noted for its prominent contribution. Further, in our data set, four groups of traits were identified in the PCA biplot considering both PC1 and PC2 simultaneously (Figure 5). The most prominent among those in group 1 were leaf lower epidermis thickness, total chlorophyll, stem metaxylem diameter, soluble sugars, and stem phloem length, while stem epidermis thickness, protein content, leaf metaxylem diameter, leaf phloem length, and width were the most prominent in group II.

## 4. Discussion

The faba bean cultivars can produce high yields, but they are highly sensitive to environmental stresses, especially drought stress. Therefore, determining suitable attributes in the wild relative to legumes for boosting legume growth and production under water-deficient conditions is crucial. In the current study, *Vicia* wild relatives were evaluated for physiological, biochemical, and anatomical attributes. Photosynthetic pigments are vital for plant normal growth and are important predictors of describing the health condition of plants, especially under stress [57]. A reduction in photosynthetic pigments is the early response of plants toward water-deficit conditions, which leads to a reduction in the final yield and accumulation of metabolites [37,58]. Concerning photosynthetic pigments, current results revealed a decrease with an increase in the severity of drought stress. Our current results support the findings of Abbasi et al. [59] who also reported a reduction in chlorophyll contents under drought stress conditions in *Vicia sativa.* A similar reduction in photosynthetic pigments under drought stress conditions was also reported by [57,60].

Soluble sugars may have a prominent role in osmotic protection, the stability of cell membranes, and turgor pressure. Therefore, variances in the accumulation of SS may be considered genetic factors which can affect plant morpho-physiological responses under water-deficit conditions [61]. The current results revealed a reduction in SS under drought stress conditions. Our results support the findings of Hammad and Ali [62], who reported decrease in soluble sugar contents with an increase in drought stress.

The accumulation of proteins such as dehydrins late embryogenesis, abundant under water-deficit conditions, indicates drought-tolerant ability of plants. However, the reduction in certain other proteins under stress conditions and the extent of decline or accumulation largely depends on a genetic variation of different plant species [63,64]. Concerning protein contents, the current study revealed a decline under water-deficit conditions. Our findings supported the results of Parida et al. [65] who also reported a reduction in protein contents under drought stress conditions. Similarly, Mansour et al. [66] also reported that protein contents were significantly reduced in the studied faba bean under drought stress conditions.

Osmotic adjustment is a vital process of plants for maintaining water uptakes and cell wall pressure under drought stress conditions [67,68]. An increase in the accumulation of proline contents under water-deficit conditions is considered to be beneficial for the osmoprotection of plants and, generally, it is recommended as the criteria for selection tolerance against drought stress [69,70]. Our findings suggested that an increase in proline contents may have improved the plant’s ability to tolerate osmotic stress and hence, is the best indicator of stress tolerance. The present study also revealed enhanced proline contents in moderate and severe drought stresses, respectively. Our finding supports the findings of Kabbadj et al. [60] who reported an increase in proline contents with an increase in drought stress conditions. Similarly, our finding is also supported by the findings of Selim et al. [71] who work on tomato and reported the same findings. Our findings were also supported by Yadav et al. [72] who reported an increase in proline contents with an increase in drought stress.

Under drought stress conditions, plants not only face turgidity loss but also suffer from oxidative stress due to the accumulation of ROS. For the removal of these ROS, plants possess antioxidant mechanisms [73]. Peroxidase (POD) and superoxide dismutase (SOD) are major enzymes that play a key role in the defense mechanism of plants against various stress conditions by scavenging H_2_O_2_ in chloroplasts [74]. In the current study, drought stress resulted in an increased antioxidative defense system, and hence we observed variably enhanced SOD and POD activities in the studied *Vicia* species. Similar findings regarding peroxidase and superoxide dismutase activity are supported by those reported by Acar et al. [75]. The current results are also in general agreement with those previously reported [60,76].

Principle component analysis is a powerful statistical procedure to reduce the dimensions of variables and to divulge constructive evidence-driven feedback from a highly correlated dataset. PCA biplot analysis has been used widely and effectively by other researchers for screening drought-tolerant cultivars of *Vicia* and lentil species [27,77,78]. Current results clearly demonstrated that correlations of a trait pair were well coordinated with the approximation of the vector angles. PCA was a better approach in the identification of species tolerant and sensitive to progressive drought stresses.

Changes in anatomical attributes of root, stem, and leaves are diverse mechanisms through which plants can tolerate the harmful effects of drought stress [79,80,81]. Drought significantly affected the various studied leaf micromorphological attributes. Concerning epidermal cell length and width, the current study revealed a decreasing trend in both moderate and severe drought stress which are in general agreement with those reported by Boughalleb et al. [79] and Haffani et al. [82]. However, comparatively, less reduction in epidermal cell length and width was observed in *V. monantha* and *V. sativa* subsp. *sativa* in both drought stress treatments. This may have contributed toward its better water retention properties and prevention of excessive loss through transpiration. Further, a relatively lower decrease in leaf upper epidermal cell thickness under drought stress conditions may be regarded as a growth promoting/sustaining factor which may further assist its adaptation in arid environments as discussed by Zhang et al. [83]. The maximum CO_2_ assimilation in drought stress environment is considered as an adaptation strategy which can be achieved if stomatal changes in plants synchronize the interrelationship between water, transpiration, and photosynthesis, with the ultimate result being reduced levels of tissue damages [84]. We noted the enhanced stomatal length and decreased width with an increase in drought stress conditions as reported by Li et al. [85]. Our findings are similar to findings of Makbul et al. [86], who also reported a decrease in stomatal width with an increase in drought stress in soybean plants, concerning trichome length and width showed a decreasing trend with an increase in drought stress. Similar findings were also reported by Yadav et al. [72] who reported an increase in trichome length with an increase in the concentration of drought stress. Chen et al. [87] also discussed the densities of leaf trichomes in relation to the availability of water and its effects on plant growth and development in terms of increased cell size and expansion in the leaf area.

There was a decreasing trend in the studied stem anatomical attributes with increasing water deficit. For instance, epidermal and ground tissue, phloem length and width, and xylem elements exhibited progressive reduction both under MS and SS. The adaptation of wild *Vicia* spp. in these traits may be used for the identification of drought-tolerant species to overcome current food security crises [88,89]. Concerning epidermal tissue thickness, the current results revealed a declining trend under drought stress environments in comparison to the control-grown plants. Boughalleb et al. [79] also reported a reduction in epidermal thickness in plants under drought stress conditions. Similarly, a significant decrease in metaxylem thickness with increase in drought stress was recorded which was in agreement with those reported by Haffani et al. [82]. Our result revealed that the decreased xylem vessel diameter, and the phloem length and width increased in drought stress. This reduction may be due to a decrease in the radius of the sieve tube and an increase in viscosity of phloem sap, which in turn may affect the capacity of phloem transport due to lesser phloem conductance in the stem as previously reported [28,90,91]. The dissection of the complex architecture of quantitative traits in drought stress environments may lead to identification of favorable SNPs and haplotypes, underpinning traits of breeding interest through crop-wild introgressions [92,93,94,95]. The findings of current research work suggest its implications for detailed molecular characterization of wild *Vicia* species which may further help researchers for its rational use in their breeding efforts. The study provided the basis for further genetic diversity studies of wild *Vicia* species through high-throughput genotyping platforms which ultimately will lead to marker-assisted selection (MAS) in breeding programs in climate change perspectives. De la Rosa et al. [96] recently conducted similar genetic diversity studies of a large collection of *Vicia sativa* accessions and reported some potential drought-tolerant accessions to be utilized as promising sources in breeding programs. Proline metabolism has been reported as among the important pathways enriched in the leaves and roots of *Vicia sativa*. In a study by Min et al. [97] related to differentially expressed genes (DEG) of sucrose non-fermenting 1-related protein kinase (SnRK) in *Vicia sativa*, two SnRK DEGs were observed to be downregulated and two upregulated in both leaves and roots, suggesting its role as signal transducers and controlling phosphorylation of stress-responsive genes in a drought stress environment.

## 5. Conclusions

Variable responses were observed for the investigated physiological, biochemical, and anatomical traits among studied *Vicia* species which may/could be considered effective indices for selection against drought tolerance in breeding. In comparison with other species, *V. sativa* subsp. *sativa, V. narbonensis,* and *V. monantha* were found most tolerant to drought stress because of a lower decrease in chlorophyll contents and soluble sugar under drought stress. These species also showed better tolerance to drought stress by exhibiting increased proline contents, and POD and SOD activities. Concerning anatomical traits, the most tolerant species were *V. sativa* ssp. *sativa, V. narbonensis,* and *V. bithynica,* which showed comparatively less reduction in epidermal thickness, stomatal length and width, stomatal complex length and width, trichome length and width, phloem length and width, and metaxylem diameter. The research work depicted the importance of *Vicia* wild species and its further utilization in breeding faba bean and other legume crops for drought tolerance. The study provided the basis for further genetic diversity studies of wild *Vicia* species through high-throughput genotyping platforms which ultimately will lead to marker-assisted selection (MAS) in breeding programs in climate change perspectives. Moreover, further research is recommended in order to investigate the underlying mechanism of drought stress tolerance at the molecular and cellular level.

## Figures and Tables

**Table 1:** Analysis of variance, mean values, and percentage changes in leaf abaxial surfaces of *Vicia* species.

Mean Squares									
Source of Variation	DF	AbECL	AbECW	AbSTL	AbSTW	AbSTCL	AbSTCW	AbTRL	AbTRW
Genotypes	5	5446.21 ***	889.642 ***	1418.11 ***	199.55 ***	15,388.1 ***	10,008.3 ***	42,349.7 ***	571.076 ***
Drought stress	2	1001.83 ***	256.722 **	125.34 **	90.546 ***	900.9 *	262.9 *	33499.1 **	247.419 ***
Drought stress x genotypes	10	80.37 ^NS^	11.804 ^NS^	23.50 ^NS^	16.975 *	146.8 ^NS^	68.8 ^NS^	1194.7 ^NS^	13.018 ^NS^
Error	153	305.42	50.84	17.88	7.9	189.8	95.7	7011.2	33.915
Mean control		85.672	33.818	29.627	20.927	86.113	66.394	410.15	28.969
Mean moderate drought stress		80.491	30.455	30.83	19.716	79.267	63.228	381.23	26.824
Mean severe drought stress		77.608	30.05	32.505	18.47	79.545	62.438	363.32	24.909
% age change due to moderate stress		6.048	9.95	−4.061	5.79	7.96	4.78	7.052	7.42
% age change due to severe stress		9.42	11.15	−9.72	11.75	7.6271875	5.9583697	11.42	14.03

AbECL, epidermal cells length; AbECW, epidermal cells width; AbSTL, stomatal length; AbSTW, stomatal width; AbSTCL, stomatal complex length; AbSTCW, stomatal complex width; AbTRL, trichome length; AbTRW, trichome width. *, ** and *** are showing statistical significance at the *p* ≤ 0.05, *p* ≤ 0.01 and *p* ≤ 0.001 probability level; NS, non-significant.

**Table 2:** Analysis of variance, mean values, and percentage changes in leaf adaxial surfaces of *Vicia* species.

Mean Squares									
Source of Variation	DF	AdECL	AdECW	AdSTL	AdSTW	AdSTCL	AdSTCW	AdTRL	AdTRW
Genotypes	5	8855.72 ***	1609.57 ***	544.44 ***	387.417 ***	4717.23 ***	6544.8 ***	188,972 ***	642.093 ***
Drought stress	2	2044.85 ***	390.59 ***	169.702 ***	81.501 ***	328.34 ^NS^	342.34 ^NS^	114,122 ***	138.921 *
Drought stress x genotypes	10	138.38 ^NS^	110.27 *	45.795 ***	5.095 ^NS^	308.54 ^NS^	27.96 ^NS^	5467 ^NS^	9.788 ^NS^
Error	153	203.18	52.07	12.724	8.548	219.7	126.51	14,022	30.787
Mean control		84.223	33.505	26.158	20.038	0.629	62.712	430.45	26.433
Mean moderate drought stress		81.826	30.82	29.099	18.898	76.638	60.06	365.41	24.073
Mean severe drought stress		73.129	28.405	29.043	17.707	76.52	57.945	347.6	23.589
% age change due to moderate stress		2.85	8.012	−11.25	5.70	4.95	4.25	15.15	8.95
% age change due to severe stress		13.18	15.24	−11.0291	11.65	5.098	7.65	19.25	10.80

AdECL, epidermal cells length; AdECW, epidermal cells width; AdSTL, stomatal length; AdSTW, stomatal width; AdSTCL, stomatal complex length; AdSTCW, stomatal complex width; AdTRL, trichome length; AdTRW, trichome width. *, ** and *** are showing statistical significance at the *p* ≤ 0.05, *p* ≤ 0.01 and *p* ≤ 0.001 probability level; NS, non-significant.

**Table 3:** Analysis of variance, mean values, and percentage changes in stem and leaf anatomical traits of *Vicia* species.

Mean Squares										
Source of Variation	DF	LUET	LLET	LPHL	LPHW	LMXD	SEPT	SPHL	SPHW	SMXD
Genotypes	5	148.03 ***	327.44 ***	5008.2 ***	366.95 ***	178.74 ***	267.79 ***	74,045.4 ***	23,322.3 ***	291.83 ***
Drought stress	2	57.78 ***	49.76 **	135.99 ^NS^	68.92 *	21.22 **	145.32 **	829.8 ^NS^	446.7 ^NS^	140.93 ***
Drought stress x genotypes	10	3.36 ^NS^	2.08 ^NS^	23.72 ^NS^	2.18 ^NS^	0.65 ^NS^	4.05 ^NS^	48.01 ^NS^	32.3 ^NS^	6.28 ^NS^
Error	153	6.79	9.13	64.68	18.84	3.31	16.53	822.5	217.1	17.93
Mean control		13.34	13.56	39.36	21.17	7.82	20.40	117.01	64.59	21.94
Mean moderate drought stress		12.05	12.47	38.01	20.04	7.09	18.61	113.45	62.06	19.94
Mean severe drought stress		11.42	11.75	36.36	19.03	6.64	17.30	109.58	59.15	18.93
% age change due to moderate stress		9.69	8.059	3.45	5.35	9.24	8.77	3.04	3.92	9.12
% age change due to severe stress		14.43	13.34	7.64	10.12	15.09	15.19	6.35	8.44	13.72

where, LUET, leaf upper epidermis thickness; LLET, leaf lower epidermis leaf; LPHL, leaf phloem length; LPHW, leaf phloem width; LMXD, leaf metaxylem diameter; SEPT, stem epidermal thickness; SPHL, stem phloem length; SPHW, stem phloem width; SMXD, stem metaxylem diameter. *, ** and *** are showing statistical significance at the *p* ≤ 0.05, *p* ≤ 0.01 and *p* ≤ 0.001 probability level; NS, non-significant.

**Table 4:** Percentage changes in studied traits of leaf abaxial surfaces in studied *Vicia* species under drought stress.

	**AbECL**					**AbECW**					**AbSTL**					**AbSTW**				
	**% Changes**					**% Changes**					**% Changes**					**% Changes**				
***Vicia* Species**	**Control**	**MS**	**SS**	**MS**	**SS**	**Control**	**MS**	**SS**	**MS**	**SS**	**Control**	**MS**	**SS**	**MS**	**SS**	**Control**	**MS**	**SS**	**MS**	**SS**
1	72.13	68.68	67.57	4.78	6.33	28.14	27.35	25.96	2.79	7.75	21.63	20.38	19.91	5.79	7.99	15.38	16.74	17.56	−8.84	−14.14
2	71.26	69.03	65.99	3.13	7.40	34.89	31.10	30.17	10.87	13.55	25.56	27.49	30.94	−7.56	−21.05	18.83	17.08	16.49	9.31	12.45
3	75.38	67.61	66.45	10.30	11.85	32.41	28.80	27.94	11.15	13.79	36.53	38.56	39.88	−5.54	−9.14	23.27	20.96	19.90	9.94	14.50
4	90.63	85.72	84.98	5.43	6.24	34.91	28.54	31.58	18.24	9.53	30.59	31.60	33.47	−3.31	−9.42	21.46	19.44	18.95	9.38	11.68
5	98.45	95.26	93.22	3.24	5.31	43.89	40.82	39.93	7.00	9.02	35.31	39.96	40.75	−13.16	−15.40	25.57	24.42	20.86	4.53	18.43
6	106.18	96.64	87.45	8.98	17.64	28.67	26.12	24.72	8.89	13.75	28.14	27.00	30.10	4.07	−6.94	21.05	19.66	17.07	6.62	18.91
	**AbSTCL**					**AbSTCW**					**AbTRL**					**AbTRW**				
	**% Changes**					**% Changes**					**% Changes**					**% Changes**				
	**Control**	**MS**	**SS**	**MS**	**SS**	**Control**	**MS**	**SS**	**MS**	**SS**	**Control**	**MS**	**SS**	**MS**	**SS**	**Control**	**MS**	**SS**	**MS**	**SS**
1	48.92	46.15	45.64	5.67	6.72	35.69	37.96	38.76	−6.37	−8.61	441.02	402.70	377.67	8.69	14.37	29.70	26.80	24.36	9.76	17.98
2	78.78	74.90	71.36	4.93	9.43	59.52	56.33	53.72	5.37	9.75	362.98	345.24	337.86	4.89	6.92	27.91	24.16	22.20	13.46	20.48
3	84.91	83.01	81.13	2.23	4.44	74.33	73.48	68.24	1.16	8.20	399.31	356.81	321.37	10.64	19.52	33.76	35.42	33.16	−4.93	1.77
4	110.27	88.82	96.72	19.45	12.29	82.80	72.03	75.89	13.01	8.34	420.90	396.65	375.24	5.76	10.85	27.46	25.64	24.46	6.64	10.94
5	117.25	111.67	109.01	4.76	7.03	90.27	86.84	86.24	3.80	4.47	470.58	433.16	426.73	7.95	9.32	31.37	28.24	26.45	9.96	15.68
6	76.55	71.06	73.42	7.17	4.08	55.75	52.74	51.78	5.40	7.13	366.11	352.81	341.07	3.63	6.84	23.62	20.68	18.84	12.42	20.24

AbECL, epidermal cell length; AbECW, epidermal cell width; AbSTL, stomatal length; AbSTW, stomatal width; AbSTCL, stomatal complex length; AbSTCW, stomatal complex width; AbTRL, trichome length; AbTRW, trichome width.

**Table 5:** Percentage changes in various traits of leaf adaxial surfaces studied *Vicia* species under drought stress.

	**AbECL**					**AbECW**					**AbSTL**					**AbSTW**				
	**% Changes**					**% Changes**					**% Changes**					**% Changes**				
***Vicia* Species**	**Control**	**MS**	**SS**	**MS**	**SS**	**Control**	**MS**	**SS**	**MS**	**SS**	**Control**	**MS**	**SS**	**MS**	**SS**	**Control**	**MS**	**SS**	**MS**	**SS**
1	63.76	61.76	60.56	3.15	5.02	26.50	24.50	24.17	7.58	8.82	24.20	26.62	28.67	−10.00	−18.48	17.69	16.02	15.32	9.46	13.40
2	84.21	77.98	68.14	7.41	19.08	27.66	25.85	24.52	6.55	11.4	22.28	25.08	27.36	−12.56	−22.81	16.64	15.93	14.77	4.26	11.25
3	67.23	67.35	65.17	−0.17	3.07	33.31	32.36	30.18	2.83	9.40	29.63	32.23	29.53	−8.76	0.33	21.89	19.20	19.35	12.28	11.63
4	115.17	112.56	97.25	2.27	15.56	42.62	41.10	40.82	3.57	4.22	25.89	26.84	23.46	−3.69	9.39	19.23	18.56	17.16	3.50	10.78
5	95.77	94.66	82.52	1.16	13.83	46.10	38.39	27.71	16.73	39.9	30.38	38.47	38.44	−26.62	−26.53	27.17	26.24	23.08	3.41	15.07
6	79.19	76.66	65.13	3.20	17.76	24.84	22.72	23.04	8.52	7.26	24.58	25.37	26.80	−3.21	−9.03	17.61	17.44	16.58	0.97	5.85
	**AdSTCL**					**AdSTCW**					**AdTRL**					**AdTRW**				
	**% Changes**					**% Changes**					**% Changes**					**% Changes**				
	**Control**	**MS**	**SS**	**MS**	**SS**	**Control**	**MS**	**SS**	**MS**	**SS**	**Control**	**MS**	**SS**	**MS**	**SS**	**Control**	**MS**	**SS**	**MS**	**SS**
1	65.01	63.19	60.92	2.81	6.30	41.23	41.82	38.17	−1.44	7.42	395.85	343.3	311.5	13.28	21.30	29.23	24.96	23.44	14.62	19.80
2	72.51	66.16	64.82	8.75	10.60	52.70	49.44	48.93	6.18	7.16	320.03	297.4	291.6	7.08	8.87	22.75	18.23	19.10	19.90	16.05
3	88.69	86.97	83.02	1.95	6.40	84.83	82.01	76.43	3.33	9.90	445.45	361.5	359.2	18.85	19.37	31.48	29.66	29.24	5.76	7.12
4	104.58	87.29	92.89	16.53	11.18	74.07	68.20	70.43	7.93	4.92	595.04	480.5	460.1	19.25	22.67	26.24	24.97	24.25	4.83	7.59
5	88.77	87.26	79.04	1.70	10.95	67.50	64.45	61.03	4.51	9.58	495.77	401.1	378.4	19.10	23.67	29.75	28.22	27.74	5.16	6.75
6	64.22	68.96	78.43	−7.39	22.14	55.95	54.44	52.69	2.70	5.84	330.56	308.7	284.7	6.61	13.88	19.15	18.41	17.77	3.89	7.22

AdECL, epidermal cell length; AdECW, epidermal cell width; AdSTL, stomatal length; AdSTW, stomatal width; AdSTCL, stomatal complex length; adSTCW, stomatal complex width; AdTRL, trichome length; AdTRW, trichome width.

**Table 6:** Percentage changes in various stem and leaf anatomical traits of *Vicia* species under drought stress.

	SEPT					SPHL					SPHW				
	% Changes					% Changes					% Changes				
*Vicia* Species	Control	MS	SS	MS	SS	Control	MS	SS	MS	SS	Control	MS	SS	MS	SS
1	17.55	14.22	12.15	18.97	30.77	93.59	90.43	88.11	3.38	5.85	49.9	48.14	47.43	3.45	4.87
2	20.30	18.68	17.70	7.96	12.81	61.57	60.01	58.44	2.52	5.07	34.5	32.72	30.83	5.10	10.58
3	22.67	20.73	19.38	8.58	14.50	98.32	95.12	92.98	3.25	5.43	47.2	45.42	44.45	3.79	5.85
4	24.05	23.19	22.36	3.58	7.04	109.56	105.98	103.5	3.27	5.52	53.6	51.65	48.86	3.61	8.81
5	19.11	17.50	16.21	8.40	15.15	213.09	206.00	198.3	3.33	6.97	107.7	102.04	96.31	5.27	10.59
6	18.75	17.37	16.02	7.35	14.53	125.96	123.19	116.2	2.19	7.76	94.7	92.42	86.99	2.45	8.18
	**SMXD**					**LUET**					**LLET**				
	**% Changes**					**% Changes**					**% Changes**				
1	20.09	19.61	17.45	2.37	13.12	13.69	11.11	10.10	18.86	26.23	11.45	9.28	8.99	18.91	21.50
2	15.64	14.13	13.58	9.63	13.12	12.05	11.41	11.03	5.27	8.45	13.91	12.32	11.62	11.47	16.47
3	23.61	22.52	21.62	4.60	8.44	15.59	13.40	12.84	14.02	17.61	16.45	15.62	13.94	5.08	15.27
4	23.86	20.11	19.95	15.70	16.38	13.53	12.40	11.80	8.36	12.77	12.41	11.38	10.70	8.25	13.75
5	24.95	21.04	20.80	15.68	16.66	16.10	15.10	14.40	6.25	10.58	18.03	17.39	17.00	3.56	5.69
6	23.51	22.23	20.18	5.44	14.14	9.11	8.89	8.34	2.39	8.42	9.13	8.84	8.27	3.20	9.36
	**LPHL**					**LPHW**					**LMXD**				
	**% Changes**					**% Changes**					**% Changes**				
1	34.79	31.27	25.70	10.12	26.14	21.41	19.88	19.46	7.13	9.12	6.80	6.29	5.17	7.47	23.89
2	40.09	39.54	38.19	1.37	4.74	20.01	19.60	18.85	2.05	5.78	7.46	6.85	6.64	8.26	11.06
3	55.67	54.99	54.45	1.23	2.19	22.04	21.41	19.43	2.86	11.85	12.45	11.12	10.79	10.69	13.33
4	34.22	33.29	32.70	2.71	4.44	24.30	23.14	22.50	4.76	7.42	7.47	6.88	6.31	7.94	15.63
5	50.68	49.61	48.11	2.10	5.06	24.69	22.42	21.42	9.19	13.24	8.31	7.48	7.19	10.04	13.58
6	20.73	19.34	19.00	6.74	8.36	14.56	13.76	12.50	5.52	14.16	4.40	3.95	3.73	10.28	15.25

SEPT, stem anatomy epidermal thickness; SPHL, stem phloem length; SPHW, stem anatomy phloem width; SMXD, stem anatomy metaxylem diameter; LUET, upper epidermis thickness; LLET, lower epidermis thickness; LPHL, phloem length; LPHW, phloem width; LMXD, metaxylem diameter.

## Data Availability

All the generated data are represented in the manuscript.
